# Deformation analysis of cement modified soft clay soil using finite element method (FEM)

**DOI:** 10.1016/j.heliyon.2022.e09613

**Published:** 2022-06-02

**Authors:** Damtew Tsige, Meaza Korita, Adamu Beyene

**Affiliations:** Department of Civil Engineering, Jimma Institute of Technology, P.O. Box 378, Jimma 47, Ethiopia

**Keywords:** Soft clay, Cement, PLAXIS 2D, Hardening model, Deformation

## Abstract

Engineers are facing problems in infrastructure construction in soft clay areas due to excessive soil settlement and low bearing capacity that leads to treacherous problems in the structure to damages and collapse. Therefore, this study aims to investigate the deformation characteristics of cement modified soft clay soilusing finite element method. Soil samples collected were conducted to determine grain size analysis, free swell, specific gravity, index property, unconfined compressive strength, one-dimensional consolidation and triaxial test. From the test results the soil of study area is classified as inorganic clay of high plasticity (CH). Deformation characteristics of cement-modified soft clay soil was analyzed by PLAXIS 2D finite element software. In the finite element analysis, a constitutive soil model, hardening soil model is used. Triaxial test and one-dimensional consolidation conducted on 9, 12 and 15 percent of soil- cement stabilization. From the laboratory result the pre-consolidation pressure increased with increasing stabilizer content. This is due to of a pozzolanic reaction taking place with cement. The pre-consolidation pressure of soft clay when soil modified for 9%, 12%, and 15% of cement is 190 kPa, 290 kPa, 320 kPa, and 340 kPa, respectively. From numerical analysis, the vertical deformation values of soft clay soil increased as the cement percentage increased. It is observed that the optimum percentage of cement stabilization is 15%, at this percentage of cement the highest shear strength parameters and, the lowest deformation occurred as compared to the amount of 9% and 12% of cement.

## 1 Introduction

In Civil Engineering, all projects are built into the ground. Thus, any construction needs a foundation with sufficient bearing capacity. In addition, this sufficient bearing capacity of the soil upon which civil engineering structures lie needs not to vary conditionally. Constructions which are built on soils in which their strength changes conditionally more sustainable to failure which may range to a different extent [1]. Soft to very soft clay soils are primarily associated with substantial difficulties. Since these types of soil are sensitive to deformations and possesses minimal shear strength, it may lead to structural damage during the execution and throughout the life of the projects. Such type of soil, needs either replacement with other a suitable soil, or treatment with suitable mechanism to attain enough bearing capacity and strength to support the load imposed upon it. Soil strength generally refers to the soil ability to support the load imposed by buildings or structures ideally without failure. Treated soft soil's behaves differently with different loads resulting in varying degrees of initial strength gain and final strength development to support foundation for buildings purposes [2, 3, 4].

Many parts of the world covered with huge amount of soft clay soil. This soil characterized as difficult soil for construction purpose. The structure built on it faces shear failure, differential settlement and low bearing capacity [5, 6]. The design, construction, and performance of civil engineering structures is based on soil materials. Due to mechanical, physical and chemical properties all the soils are not suitable for construction work. Therefore, based on their characteristics soils are designated as several types, and one of which is soft clay soil. . Soft clay soils have relatively low stiffness and a high level of deformation when a load, has applied on it. The deformation of soft clays is high due to its relatively low stiffness. Therefore, the deformation analysis should consider soft clay as a large-deformation continuum approach [1].

When soil is exposed to a certain amount of load it tends to deform in the direction of the application of the load. The type and value of deformation differ from one soil to another.

Soft clay soils tend to deform differently from hard soils under the same load. The deformation properties of soil depend on the origin of the soil, the structure of the soil particles, the bonds between the particles, the water content of the soil, and so on [7, 8, 9]. Soils with characteristics of low strength and high compressibility exist all over the world. One of the most significant problems that arises because of its characteristics are problems in supporting loads on such a foundation. The stability of structures faces difficulties when constructed on soil with low strength [9, 10].

Soil is established from soil particles, and pore system between soil particles. The Pore system can be filled by water, a small amount of water, or without water. Compressing the soil with low or no moisture causes the first soil skeleton to deform quickly, then the chains between the soil particles to break, causing the soil particles to move closer to each other. As a result, the porosity and formation gradually decrease and the soil becomes denser. For saturated soil when soil is compressed, the behavior is almost the same as that of the above mentioned. However, the pore is filled by water, the soil particles move closer to each other and the water in the pore is dissipated. Compressibility of saturated soil associated with pore water dissipation is called consolidation. The consolidation process can be divided into two stages such as primary consolidation which is the process that water in pore dissipates, the pore becomes smaller, thus the soil is denser and the other is secondary consolidation that is the process by which pore water has fully dissipated, however, soil particles continue moving, and sliding over each other to a stable position. Therefore, the stabilization of soil is important to be done in the construction. The aims of soil stabilization is to enhance the strength and durability of soil which can be increase the engineering performance of soil... Therefore, soil-aditives mixe improves the density of soil, stiffness, compressibility, permeability, workability and chemical and physical properties of soil [11, 12, 13, 14].

Soil behavior under load application is complicated. Soils may change from idealized linear homogenous and isotropic behavior to nonlinear heterogeneous and anisotropic behavior when subjected to stresses. The selection of an appropriate soil constitutive model is essential for successful finite element analysis (FEA). FEM allows for easier modeling of complex geometrical and irregular shapes. It adapted to meet certain specification for accuracy in order to decrease the need for physical prototype in design process. FEM can solve the problem with high degree of accuracy. The model is highly useful for time dependent simulations soil consolidation and deformation analysis. Even though, numerous constitutive models have been proposed to describe a various aspects of soil behavior in FEM, none of the available constitutive models can reproduce all aspects of soil behavior. Therefore, whatever model is chosen, its predictive capacity comes from the comparing with field observations [15].

The utilization of numerical analysis in solving engineering problems is based on the actual mechanical behavior of soil material represented by constitute model. The model related with particular soil material has to describe material change under external load application. The stress-strain behavior of soil can be represented with variety of models [16]. Due to the complexity of real soil behavior, a single constitutive model that can describe all facets of behavior, with a reasonable number of input parameters, has not been achieved. Consequently, soil models are continuously developed and improved; hence there are many soil models available today, each of which has different advantages and limitations.

The linear elastic model of soil is based up on linear elasticity of hooks law. This model consists of two basic model parameters these are modulus of elasticity (E) and poisons ratio μ. The Linear elastic soil model does not consider the shear strength of the soil mass; hence over-stressing may be encountered. This is one of its main drawbacks. For this model type, there is no defined yield value and computed strains may be very unrealistic. Using cohesion and friction angle along with Mohr-coulomb failure criterion will enable the computed shear stress to be compared visually with theoretical yield stresses. This model is limited for the simulation of soil behavior. It is confined to stiff structures in the soil mass. Soil by nature is highly nonlinear and irreversible hence the important characteristics of soil not sufficiently described in linear elastic soil model [17]. Elastic-plastic behavior of soil modeled by Mohr-Coulomb model. The stress-strain of soil behaves linearly in elastic range which governed by stiffness parameters of soil namely Young's modules, E and Poisson's ratio,ν. The failure criteria and flow rule of soil is governed by shear strength parameter (the friction angle, φ and cohesion, c) and dilatancy angle, ψ respectively. Thus this model can be applicable in shallow foundations, dam and slope stability, and embankment stability [17].

Modified cam clay is an elastoplastic strain hardening model that uses hardening plasticity to model non-linear behavior. This model is based on the criticality theory and basic assumption that there is an algorithmic relationship between the average and effective stress and the porosity. The original and compressed lines are linear in space. This is most realistic with almost normally consolidated clay. Only linear elastic behavior is modeled before yielding and may result in unreasonable values of ν due to log-linear compression lines. This model is more suitable for deformation than failure, especially for normally consolidated soft soils. The above limitations were confirmed by [18].

Nonetheless, this model is an essential feature of the behavior commonly observed in non-drainage tests of loose sand and normally consolidated, undisturbed clay, which is the peak deviation stress before reaching criticality. Criticality conditions were less successful in modeling granular materials, as observed softening and dilatency of dense sand and non-drainage response of very loose sand could not be predicted. The above limitation have been confirmed by [18] and the materials modeled by critically model appear to be predominantly limited to saturated clay and silts, and hard over consolidated clays are common to critically formulations. The soft soil model is based on the modified cam clay model, but some of its drawbacks have been improved in this model. First of all, the model does not involve the over-prediction of the shear strength for over-consolidated states of stress. In fact, a Mohr-Coulomb failure criterion has been added to improve the model's capabilities of the model at failure. The “friction constant” *M*, which determines the steepness of the critical state line, and in addition to that the $k_{o}$-value can be obtained. The stiffness behavior in the soft soil model is obtained from the logarithmic relationship between the mean effective stress, *p*, and the volumetric strain, εv (rather than the void ratio). Therefore, this model slightly modified parameters $\lambda^{*}$ and $k^{*}$ instead of the original cam-clay parameters and requires no input on the initial void ratio. The model has no advantages over the Mohr-Coulomb model in unloading problems, such an excavations or tunnel problems. Furthermore, the model can be considered a second-order model, at least for near-normally consolidated clays for the ‘loading’ type of applications mentioned above. The soft soil model does not have the capabilities to model anisotropic strength and stiffness generally observed for peat. Nevertheless, it could be used for peat as long as anisotropy does not play a major role in the application [19]. The Hardening Soil model is a true secondary model of common soils (soft and hard soil types) for all types of applications [19].

The model includes friction hardening for plastic shear strains under deviation loads and cap hardening for modeling plastic volumetric strains under primary compression. You can distinguish between the two main types of hardening: shear hardening and compression hardening. Shear hardening is used to model irreversible strain due to primary deviation loads. Compression hardening is used to model irreversible plastic strain due to primary compression under odometer and isotropic loads. The current model includes both types of curing. Failures are defined using Mohr-Columb failure criteria. With two types of hardening, this model is also suitable for problems with reduced average stress due to simultaneous recruitment of shear strength. Such situations occur in excavation and tunnel construction projects. Some basic characteristics of model are stress dependent stiffness according to a power law (m). Plastic straining due to primary deviatoric loading ($E_{50}^{ref}$), plastic straining due to primary compression ($E_{oed}^{ref}$), elastic unloading/reloading input parameters ($E_{ur}^{ref}$) and failure criterion according to the Mohr-Coulomb model (*c* and φ). Enhancement of engineering properties of soft clay soils, and deformation analysis were studied. Moreover, different authors used different stabilizing agents for the investigation of deformation analysis with different percentages of stabilizers. This indicates that soft soils are highly influenced by the construction of any foundation. Additionally, most studies used Mohr-Coulomb soil model for simulation of soil behavior [20] but, Mohr- Coulomb soil model is its limitation which is used a few parameters for simulation purposes [21] used experimental study on deformation analysis. In addition [22]. It was suggested that improving the properties of soft soil by stabilizing and simulating the deformation properties of soft clay soils in specific areas has not been studied. Therefore, this study mainly focuses on the deformation characteristics of cement-modified soft clay using a hardening soil model using the finite element method.

## 2 Material and methods

### 2.1 Study area

**Figure 1:**
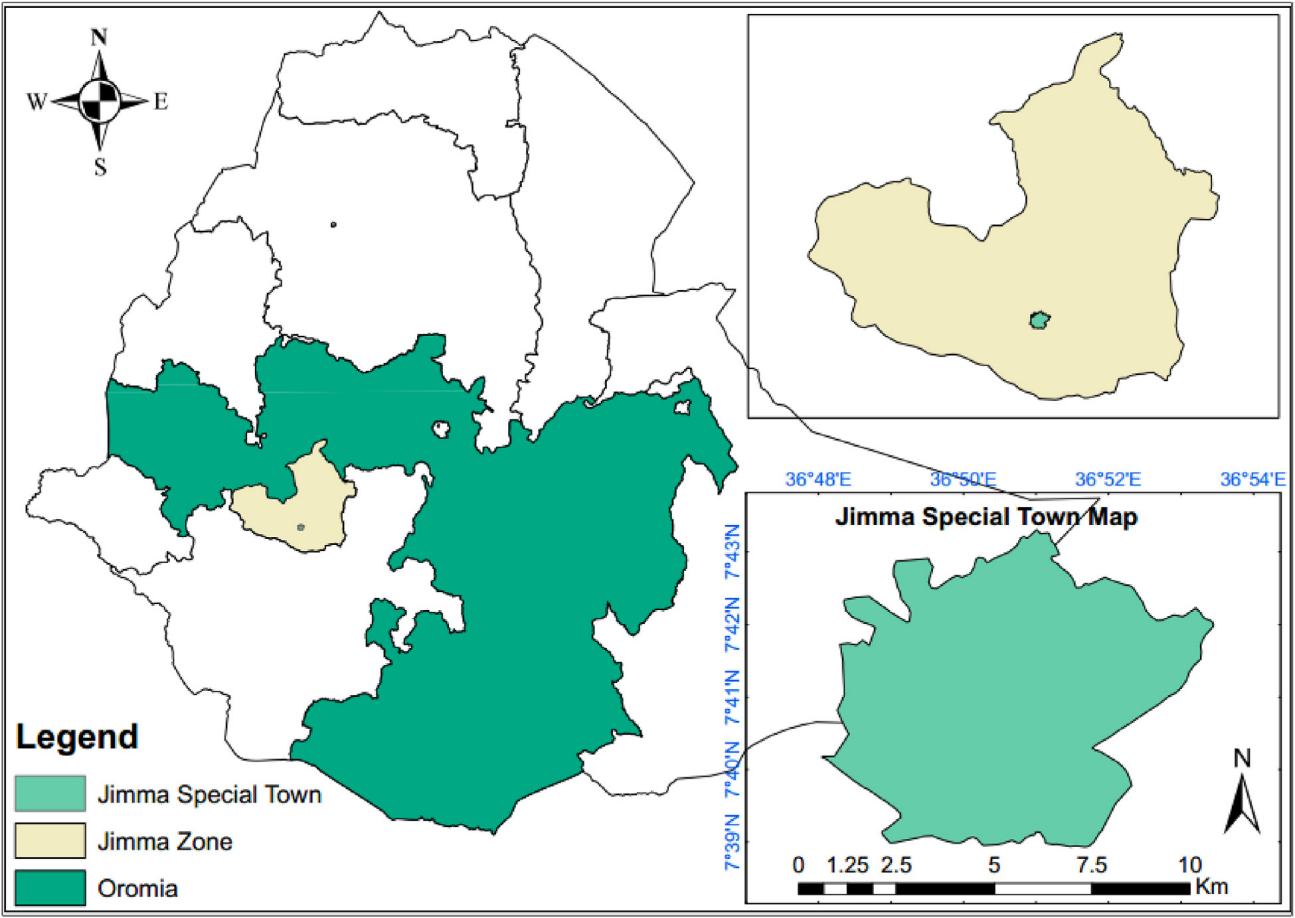
Map of the study area.

Town of Jimma is located in the southwestern part of Ethiopia in Oromia regional state. is found at a distance of approximately 352 km from Addis Ababa, the capital city of Ethiopia. The study area is located at geographical locations of 7° 13′- 8° 56 N latitudes, and 35°49′-38°38′E longitudes. The town, is located at an elevation ranges to 1718–2000 m above the mean sea. According to the recent information the population of Jimma town is 207,573 in 2020. It found in temperate the climatic zone which, is considered suitable for agriculture and human settlement. The map of the study area is shown in Figure 1.

### 2.2 Geology of the area

The town of Jimma is found in the climatic zone, which is categorized under a temperate zone, very suitable for agriculture and human settlement. The area is dominated by Bogy with soft clayey soil of quaternary alluvial deposit. They form thick loose soil of clayey-silty material that is not consolidated. The color is mostly light grey, covered with grass or low vegetation such as reeds and bogs. The northeast and southeast of the town covered by middle trachyte. In most of its revelation, the middle trachyte flows are fine to medium and light grey.

### 2.3 Materials

Cement is the most common hydraulic binder used to stabilize soils. It can be used in virtually any soil and greatly improves geotechnical properties. Studies have shown that when mixed with the cement itself and granular slag of the blast furnace. Best stabilization results in soils with high organic matter content [23]. Portland cement can be used for soil improvement or modification of soil which improved strength and durability. Portland cement it is composed of calcium silicate and calcium aluminates and hydrates to form cementitious products [24] the Chemical composition of cement is shown in Table 1.

**Table 1:** Chemical composition of ordinary Portland cement.

Constituent	$SiO_{2}$	$Al_{2}O_{3}$	$Fe_{2}O_{3}$	CaO	MgO	$SO_{2}$
Percentage	19.03	6.94	1.73	67.31	1.07	1.14

Cement is used to bind soil particles to increase the strength and stiffness of soft soil. Stabilization is achieved by mixing an appropriate amount of dry or moist cement with a certain amount of soft soil. The increase in strength of cement-stabilized soil obtained from the physicochemical reactions between the soil and cement, such as hydration of cement and the interaction between the substances in the soil and the products of hydration of cement [25] evaluated experimentally ranges of percentages of cement by weight to be tested initially for expansive soil.

### 2.4 Soft clay soil-cement preparation

Soft clay soil specimens’ preparation were extracted from Shelby tubes, and put in a standard laboratory mixer to remold the soil for about five minutes at natural moisture contents. The cement mixed with water to form a slurry using water cement ratio of 0.25. Then the cement slurry is added to soft clay soil, and further mixed until the mix becomes homogenous. Percentages of Cement used to stabilize soft clay soil are 9%, 12% and 15% by weight. Hydraulic jacks are used to compress soft clay soil. The compression mold was then placed in a custom-made extruder and a hydraulic jack was used to extrude the compressed sample vertically from the mold. Laboratory tests of cementitious clay soil mixtures performed for both one-dimensional consolidation and triaxial tests to determine the soil parameters used for numerical simulations.

### 2.5 Model geometry

**Figure 2:**
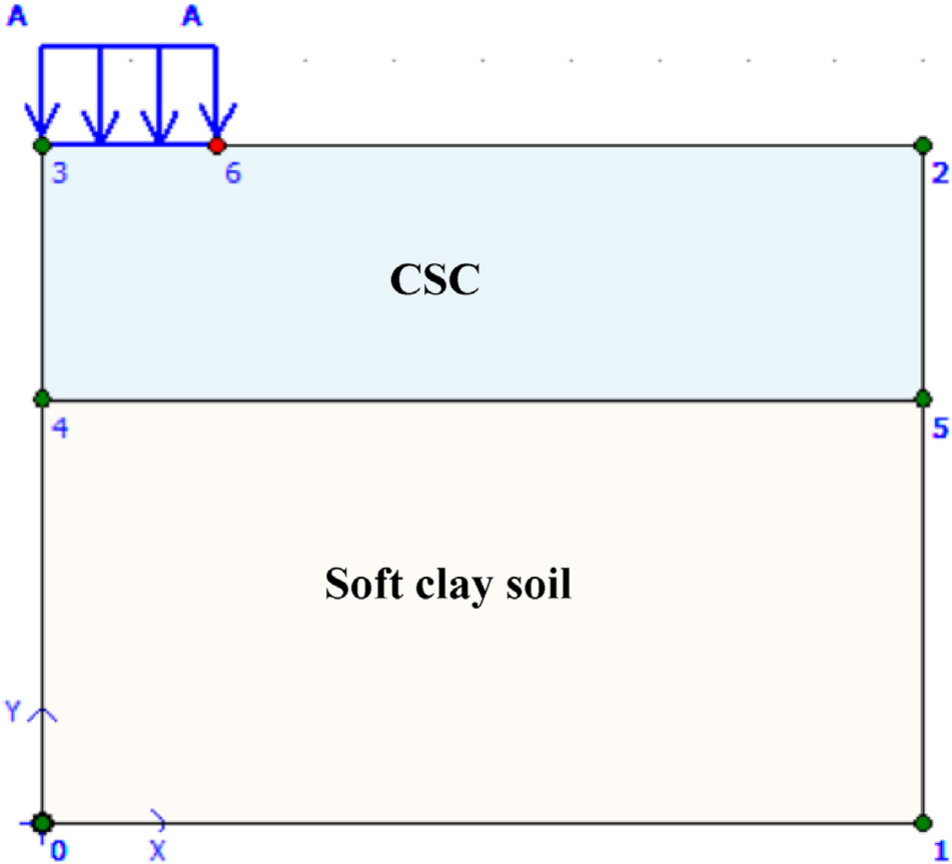
Geometry model.

Generating a finite element model using the Plaxis consolidation model begins with a geometry model. Creating a geometry model begins with drawing the geometry. In addition, materials, loads, and boundary conditions were specified and various additional loads (100, 200, 300, 400, and 500) kPa were applied to the top of the foundation (square feet) in dimensions (2 m × 2 m). Once the geometry model is created, need to compile a dataset of raw and treated soil material parameters. The depth of the soil is 8 m, and as shown in Figure 2, there is a homogeneous layer of stable soil in soft clay soil. The upper layer up to three meters is cement stabilized clay (CSC), and the second layer is soft soil.

### 2.6 Boundary conditions

**Figure 3:**
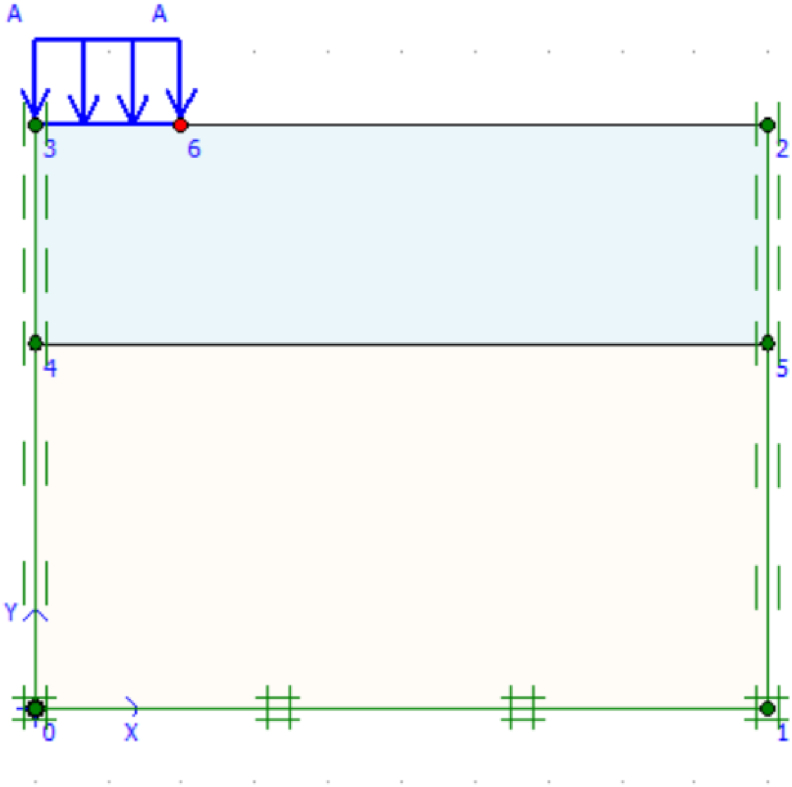
Boundary conditions.

In finite element analysis, boundary conditions are necessary to solve a boundary value problem. Lateral and bottom boundaries were set at 10 m horizontally and 4B vertically were used, B is the width of the footing. Therefore, on the bottom side, both the vertical and horizontal components of the displacement are fixed (Ux = Uy = 0), besides, the two vertical boundary edges of the geometry were set free to the vertical displacement and zero horizontal displacements (zero x displacement). The finite element analysis of boundary conditions is shown in Figure 3.

### 2.7 Discretization (Meshing)

**Figure 4:**
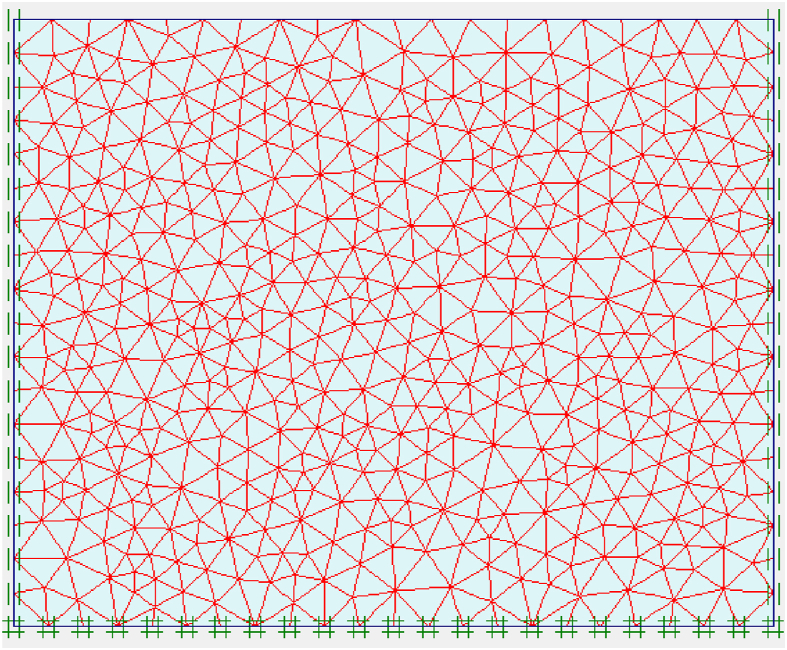
Mesh of the model geometry.

Discretization or meshing, along with the definition of material properties and boundary conditions, is a fundamental aspect of finite element modeling. Mesh size is a major contributor to the accuracy and accuracy of finite element analysis. In this study, a finer triangular mesh was used. The foundation section was considered a 2D plane strain problem and 15 node structural elements were used in the analysis. Figure 4 shows the detailed model discretization.

### 2.8 Determination consolidation settlement

Pre-consolidation stress is the boundary between the regions of small strain (rigid deformation) and large strain (soft deformation) in the void ratio log of the effective normal stress curve of the odometer test. If the final effective normal stress is less than the pre-consolidation stress, the loaded soil will experience small subsidence in a small expansion region (recompression) region. On the other hand, if the final vertical effective stress is greater than or equal to the pre-consolidation stress, the soil will experience large subsidence in areas of large strain. Another important parameter to consider is porosity. The larger the change in void rate, the higher the index and the larger settlements in both regions [26].

Before estimating the settlement, it is necessary to find out the possible region of compression of soil in question. The stress history of the soil (over-consolidated or normally consolidated) should be established. This is obtained by computing the consolidation ratio (CR) and then choosing the appropriate formula for estimating the consolidation settlement. The *CR* of soil is given as a ratio of effective pre-consolidation pressure, and the effective present overburden vertical stress, the ratio of effective pre-consolidation pressure determine by Eq. (1).

(1) $$CR=\frac{\sigma_{p}^{\prime }}{\sigma_{v0}^{\prime }}$$

Where; $\;\sigma_{p}^{\prime }$ = pre-consolidation pressure, kPa, $\sigma_{v0}^{\prime }$ = initial effective vertical stress, kPa. It is required that the *CR* is computed based on past pressure, but due to some reasons such as erosion or excavation, this past pressure cannot be quantified and, therefore, the pre-consolidation pressure is used to compute the *CR* regardless of the cause of pre-compression [27]. If the *CR* is between 0.8 and 1.2 the soil is categorized as normally consolidated (NC) soils. The soil at this transition point is more likely to experience a large strain. Therefore, the analytical computation for primary consolidation settlement is done on the region of large strain.

### 2.9 Consolidation settlement of normally consolidated soil

The pre-consolidation pressure of normally consolidated soil is approximately equal to the final effective normal stress at the point of interest in the compressible formation. Therefore, consolidation settlement in the large strain region is calculated using the change in porosity under the compression virgin gradient. According to [28], this magnitude is given as a product of void ratio and thickness of the layer, which is inversely proportional to a specific volume, ν given by Eq. (2)

(2) $$\delta_{c}=\frac{\text{Δ}e_{i}}{v}H_{0i}\;$$

Where: δ*c* = the settlement at the end of primary consolidation, m, *Hoi* = thickness of sub layer, Δ*ei* = change in void ratio and ν = specific volume.

The specific volume for saturated soils is given by Eq. (3).

(3) $$v=\frac{v_{total}}{v_{s}}$$

Where: *V total* = Total volume, which is the sum of the volume of water (*Vw*) plus a volume of solid (*Vs*).

### 2.10 Consolidation settlement of over-consolidated soil

Consolidation settlement analysis of over-consolidated (OC) soils falls into two categories. The first category is when OC soil experiences a loss of overload stress and the final vertical effective stress is less than the pre-consolidation pressure. This means that at some point in the past, the soil was exposed to a stress equal to the pre-consolidation stress, after which the overload stress decreased for some reason (erosion, drilling, landslides, etc.). However, the pre-consolidation pressure does not change. Therefore, a small deformation is expected. Therefore, the settling of the cure can only be calculated on the small strain side with the recompression gradient Cr., the consolidation settlement can be determine by Eq. (4).

(4) $$\delta_{c}=\sum \left[\frac{c_{ri}}{1+e_{0i}}H_{0i}\;\log\left(\frac{\sigma_{vfi}^{,}}{\sigma_{v0i}^{,}}\right)\right]$$

Where, $H_{0i}$ = thickness of sub-layer (m), $\sigma_{vfi}^{,}$ = final effective vertical stress, kPa, $\sigma_{v0i}^{,}$ = initial effective vertical stresses before foundation loads, kPa.

In case of final effective vertical overburden stress greater than pre-consolidation pressure, the total settlement can be computed from the region of small strain to the region of a large strain of change in void ratio is calculated using Eq. (5).

(5) $$\delta_{c}=\sum \left[\frac{c_{ri}}{1+e_{0i}}H_{0i}\;\log\left(\frac{\sigma_{p}^{.}}{\sigma_{v0i}^{,}}\right)+\frac{c_{ci}}{1+e_{0i}}H_{0i}\;\log\left(\frac{\sigma_{v0i}^{,}}{\sigma_{p}^{,}}\right)\right]$$

## 3 Result and discussion

### 3.1 Experimental work results

**Figure 5:**
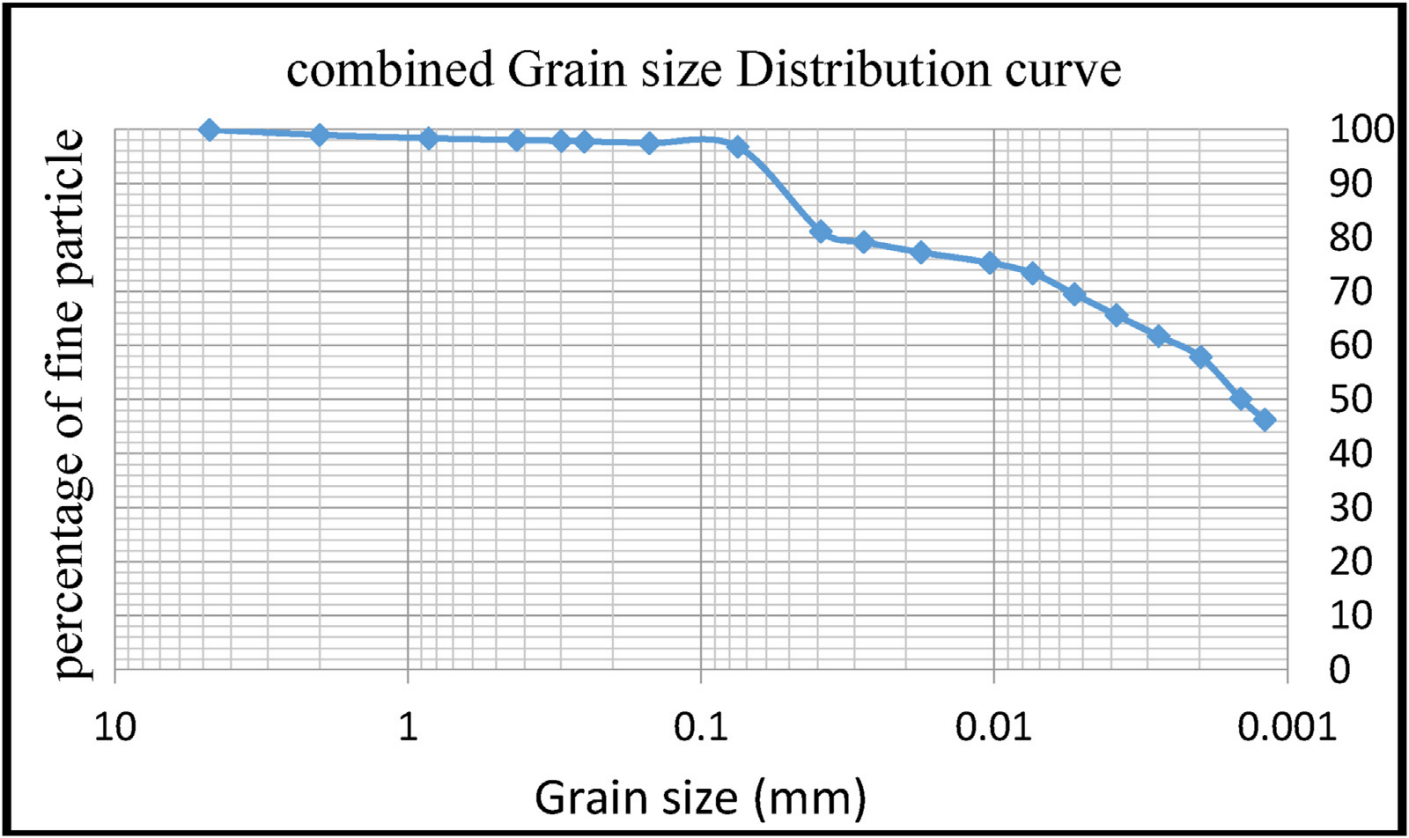
Grain size distribution curve for soft clay soil.

The test was carried out on non-drained soft clay soil supplied from the city of Jimma, Ethiopia, to determine the physical and mechanical properties of the cement before it was mixed with the soft clay soil. The results of the particle size analysis show that more than 85% of the total mass passes through a sieve size of 75 μm. This indicates that the sample is a fine-grained soil. According to hydrometer analysis, sizes above 45% are less than 5 μm (clay content according to ASTM restriction criteria). Unused soft clay has a liquid limit of 74%, a plasticity limit of 36%, and a plasticity index of 35%. According to the unified soil classification system (USCS) the soil is classified as highly plastic clay (CH). The physical properties of soft clay soil are summarized in Table 2 and Figure 5.

**Table 2:** Geotechnical properties of expansive soil for one test pit.

Soil Sample		Value	Test procedure
Moisture Content (%)		50.18	*ASTM D 4643-00*
Free swell test (%)		38	IS 2720 part 40
Specific Gravity		2.67	ASTMD792
Atterberg Limit	LL (%)	71	ASTMD4318
	PL (%)	36	ASTMD4318
	PI (%)	35	ASTMD4318
Consolidation test,KPa		190	ASTMD2166-00

### 3.2 Consolidation test

Naturally deformed samples were stabilized with a one-dimensional consolidation test Performed to get the compression parameters from the void ratio vs. applied pressure curve. The Parameters such as log-compression index Cc, swelling index Cs, pre-consolidation stress, pc, and initial porosity. The pre-consolidation pressure of the soil is defined as the highest stress the soil has ever experienced in its history. This is the pressure at which major structural changes occur, such as breaking inter-particle bonds and inter-particle displacement [29].

**Figure 6:**
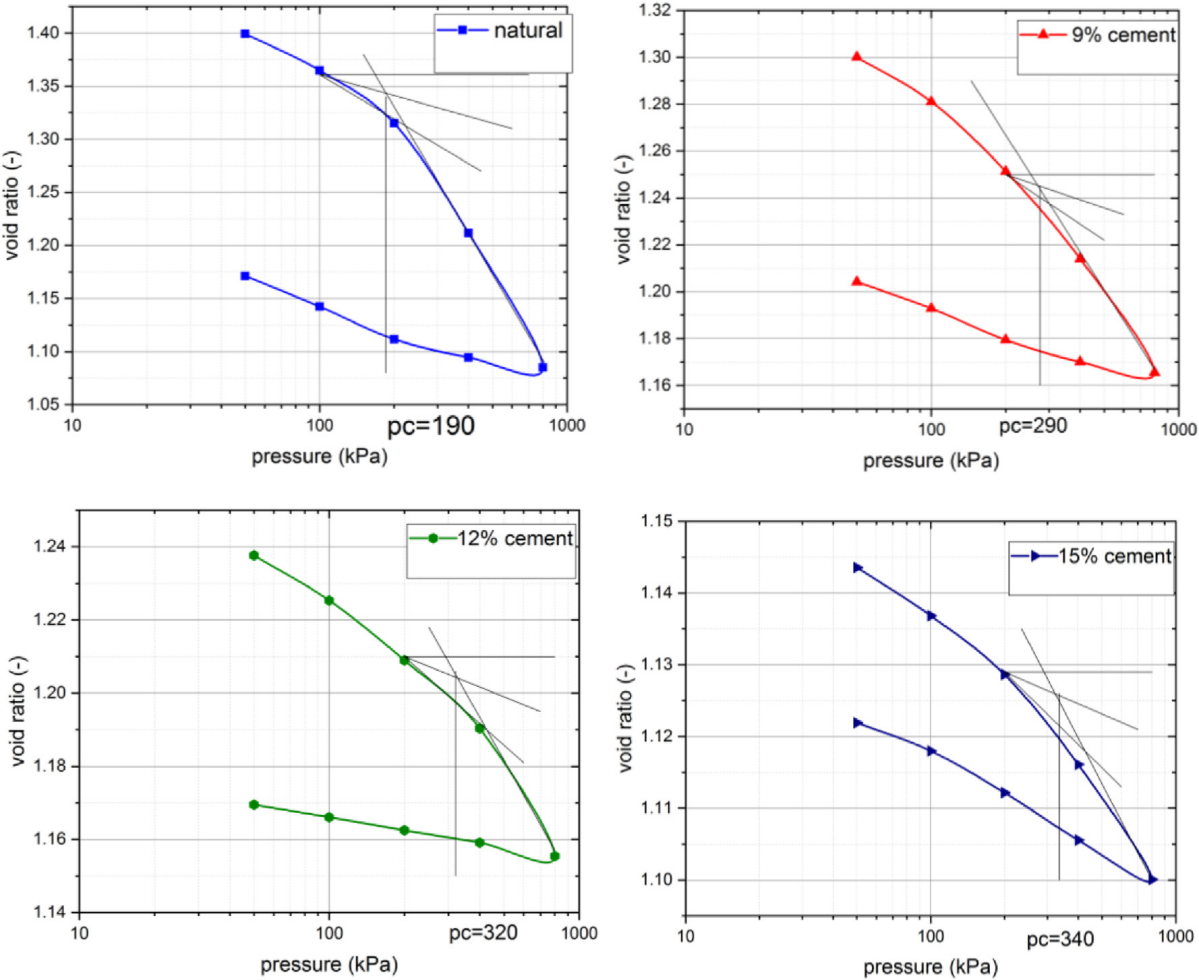
Pre-consolidation pressure, void ratio-pressure for: (a) natural soil, (b) 9% cement, (c) 12% cement, (d) 15% cement.

The practical importance of pre-integrated loads can be seen in the subsidence calculation of structures. Effect of cement addition on soft ground on consolidation pre-pressureis shown in Figure 6. The consolidation pressure value soft clay soil changed from 190 kpa to 290 KPa, 320 KPa and 340 KPa for 9%, 12% and 15%, cement-soft clay soil, respectively. It is observed that the consolidation pressure value of cement-soft clay soil treated maximum at 15% of cement. The compression and swelling indices of untreated and treated soils are varied from 0.33 to 0.07 and 0.069 to 0.016, respectively. This indicates decreases in the compressibility index and swelling indices as cement stabilization increases. Consolidation test results which are used for deformation analysis are shown in Table 3.

**Table 3:** Consolidation test result for natural and blended soft clay soil.

Cement (%)	Initial void ratio ($e_{o}$)	Cc	Cs	Pc (Kpa)
0%	1.43	0.33	0.069	190
9%	1.29	0.22	0.03	290
12%	1.24	0.12	0.02	320
15%	1.2	0.07	0.016	340

### 3.3 Triaxial test result

**Figure 7:**
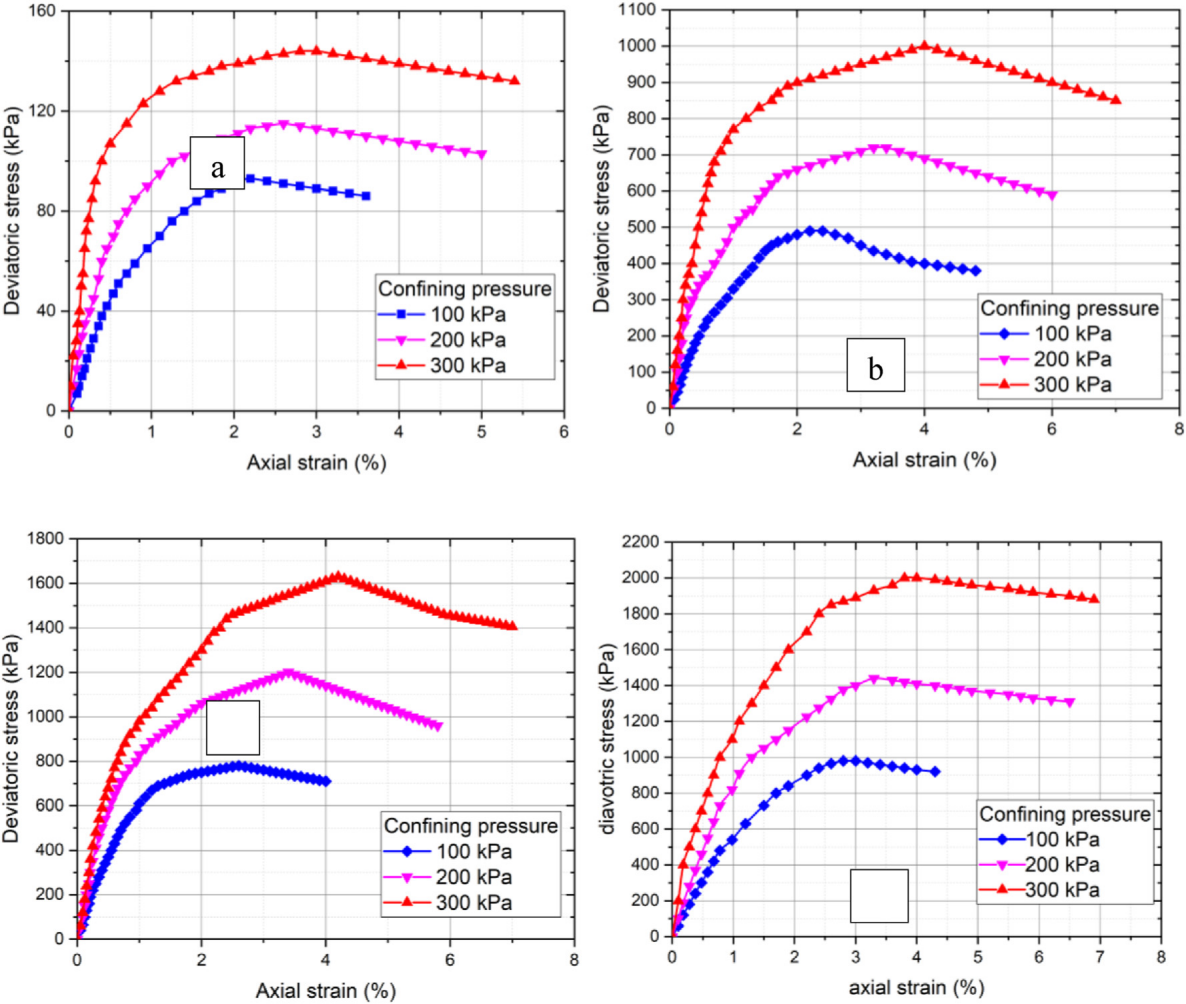
Triaxial test result of stress-strain curve for: (a) natural soil, (b) 9% cement, (c) 12% cement, (d) 15% cement.

The shear strength parameters obtained from the triaxial test for untreated and cement-treated soil are shown in Table 4. These hardening soil parameters are used in finite element analysis. Figure 7 shows an increment in shear stress for soft clay soil stabilized with cement. The un-consolidation undrained shear strength of soft clay was experimentally determined before and after the soft clay soil is stabilized with different cement percentage. The results of the experimental work were used to simulate the behavior of foundation soil using 2-D finite element model. It is observed that as the percentage of cement increases shear strength and stiffness parameters of soft clay soil increased.

**Table 4:** Un-consolidated un-drained triaxial test result.

Parameters	Natural soil	9%	12%	15%
c	29.30	63.53	68.45	75.42
φ	6.52	35.1	38.5	39.52
ψ	0	3	5.85	6.41
$E_{50}^{ref}$	3883	42,187.5	66,382.3	90,266.6
$E_{oed}^{ref}$	3883	42,187.5	66,382.3	90,266.6
$E_{ur}^{ref}$	11500	126,562.5	199,147	270,800

## 4 Finite element analysis

**Figure 8:**
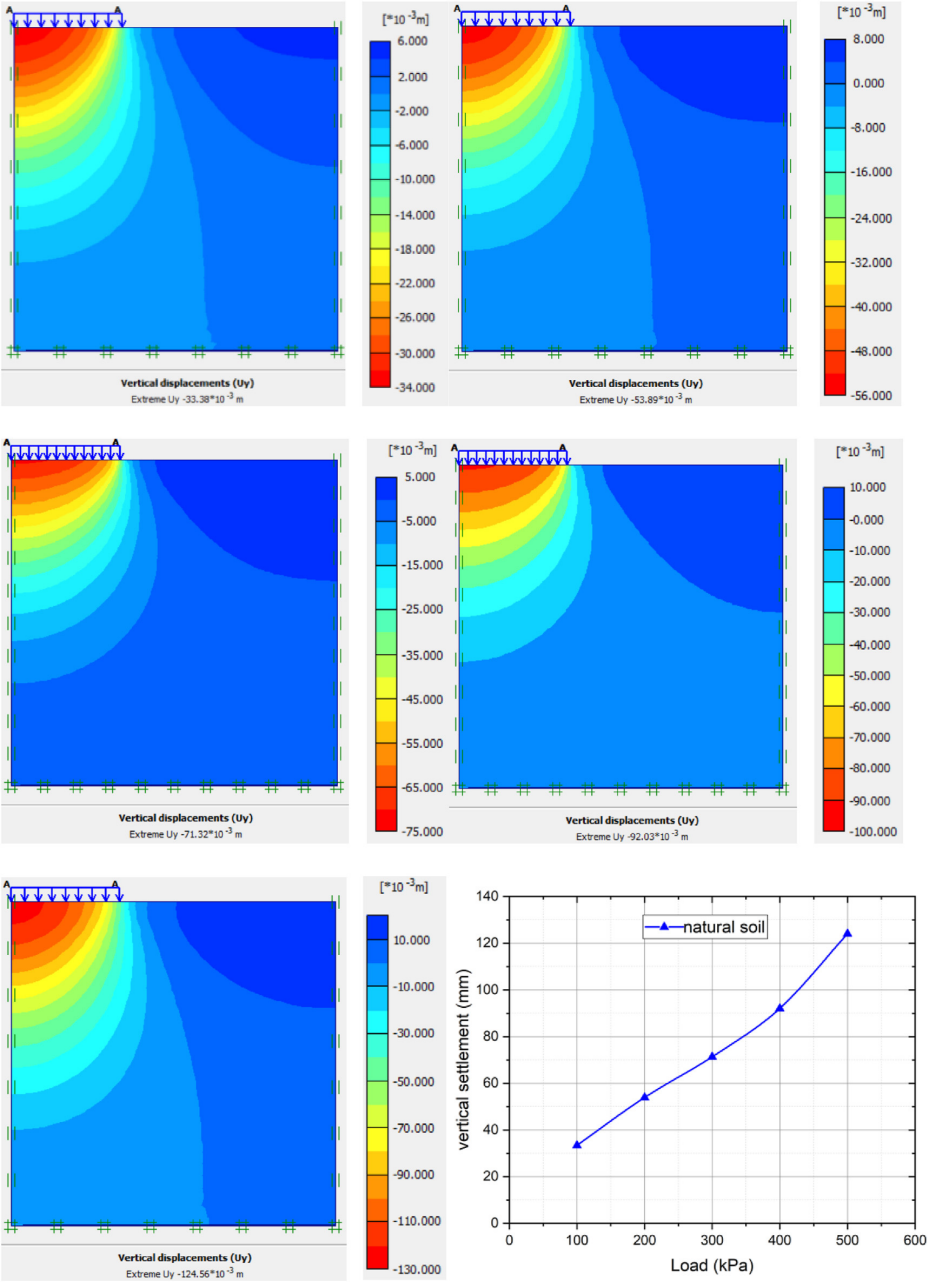
The load-settlement curve of natural soil.

Vertical displacement of natural soft clay by adding different loads in finite element analysis shown in Figure 8. The final settlement value did not increase linearly, so the soil could not absorb this load and was very compacted. The reason is that the soil is soft and has low bearing capacity Foundation settlement was predicted for different loading ranges from 100 KPa to 500 KPa using soil hardening model method. The settlement for untreated soft clay soil for 100 kpa, 200 kpa, 300 kpa, 400 kpa and 500 kpa were 33.38 mm, 55.89 mm, 71.32 mm, 92.03 mm and 124.6 mm respectively. The result revealed that, for soft clay soil, foundation deformation increased as the surcharge load increased.

**Figure 9:**
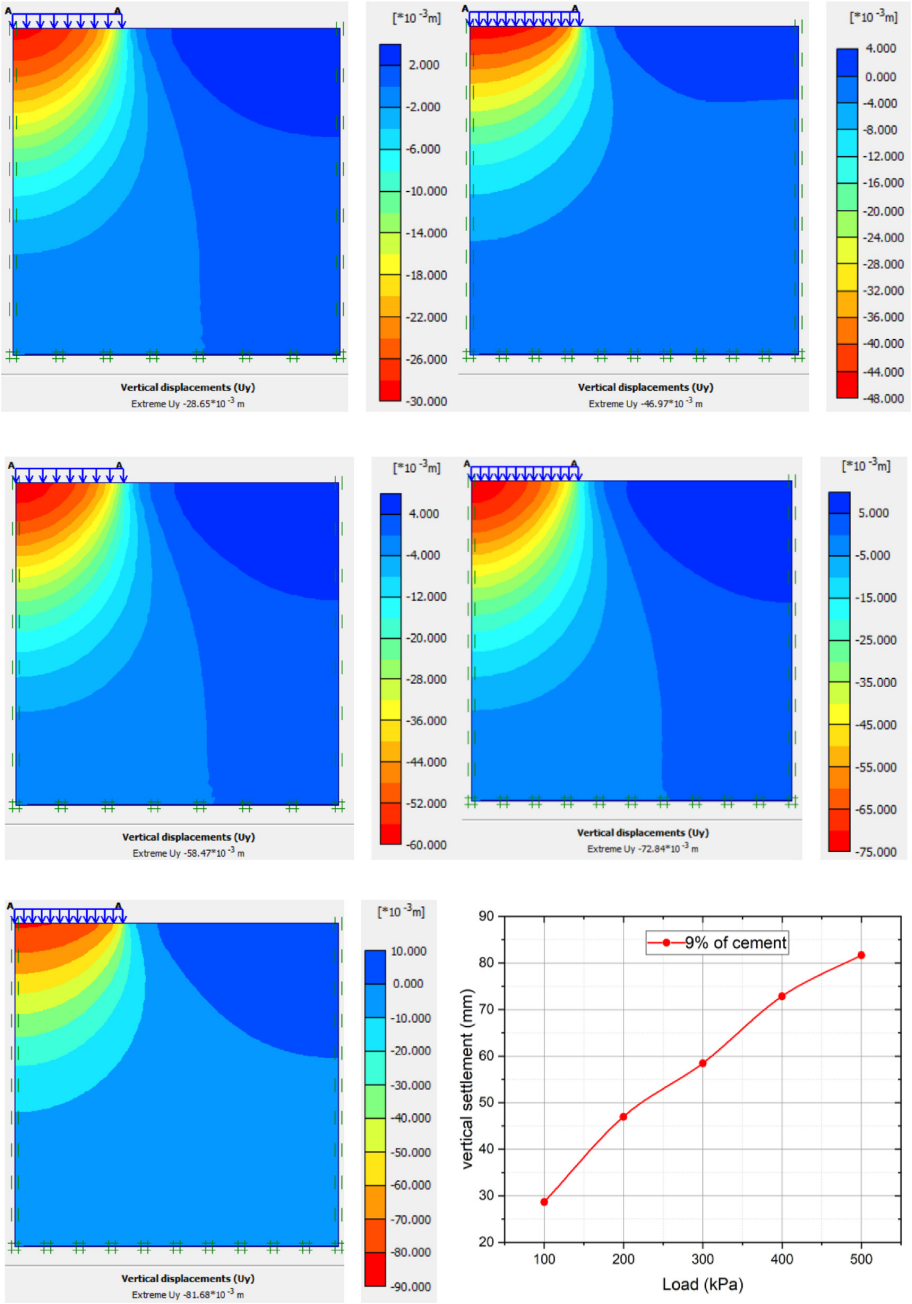
The load-settlement curve of 9% of cement stabilized soft clay soil.

Figure 9 and Table 5 show the relationship between settlement and load under 9% of cement stabilized soft clay soil. From the graph, as increasing the applied load, the settlement also increases, but when compared to natural soft clay, the magnitude of the vertical settlement in the final applied load is 81.68 mm. The average settlement 9% cement modified clay soil for 100 kpa, 200 kpa, 300 kpa, 400 kpa and 500 kpa increased by 14.14%, 15.96%, 18.01%, 36.47%, and 34.42%, respectively. It is also clearly showed that the graph is increasing in linear scale with low values settlement due to soil-cement reaction.

**Table 5:** Summary of settlement variation of applied load with various percentages of cement modified clay soil.

Cement %		0	9%	decrement in %	12%	decrement in %	15%	decrement in %
load (KPa)	100	33.38 mm	28.65 mm	14.14	9.91 mm	70.31	5.63 mm	83.13
	200	55.89 mm	46.97 mm	15.96	22.99 mm	58.86	12.18 mm	78.20
	300	71.32 mm	58.47 mm	18.01	33.38 mm	53.20	17.30 mm	75.74
	400	92.03 mm	72.84 mm	36.47	44.13 mm	52.05	22.99 mm	75.01
	500	124.6 mm	81.68 mm	34.42	60.97 mm	51.05	26.27 mm	78.91

**Figure 10:**
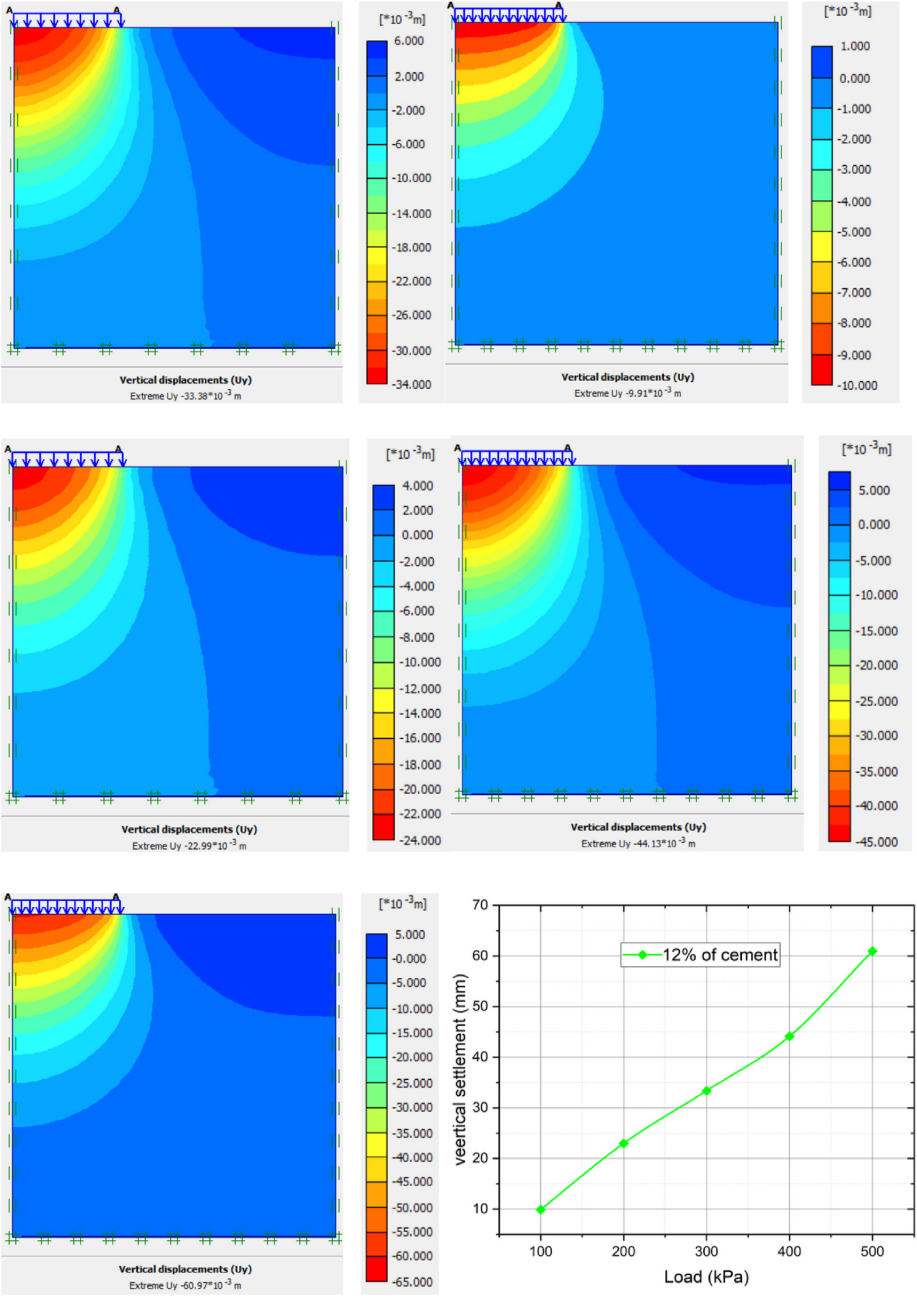
The load-settlement curve of 12% cement stabilized soft clay soil.

Figure 10 and Table 5 illustrate below the results extracted from PLAXIS software for 12% of cement stabilized soft clay. The average settlement 12% cement modified clay soil for 100 kpa, 200 kpa, 300 kpa, 400 kpa and 500 kpa increased by 70.31%, 58.86%, 53.20%, 52.05%, and 51.05%, respectively. The settlement values were less due to the high value of bearing capacity as compared to 9% of cement stabilized clay soil.

**Figure 11:**
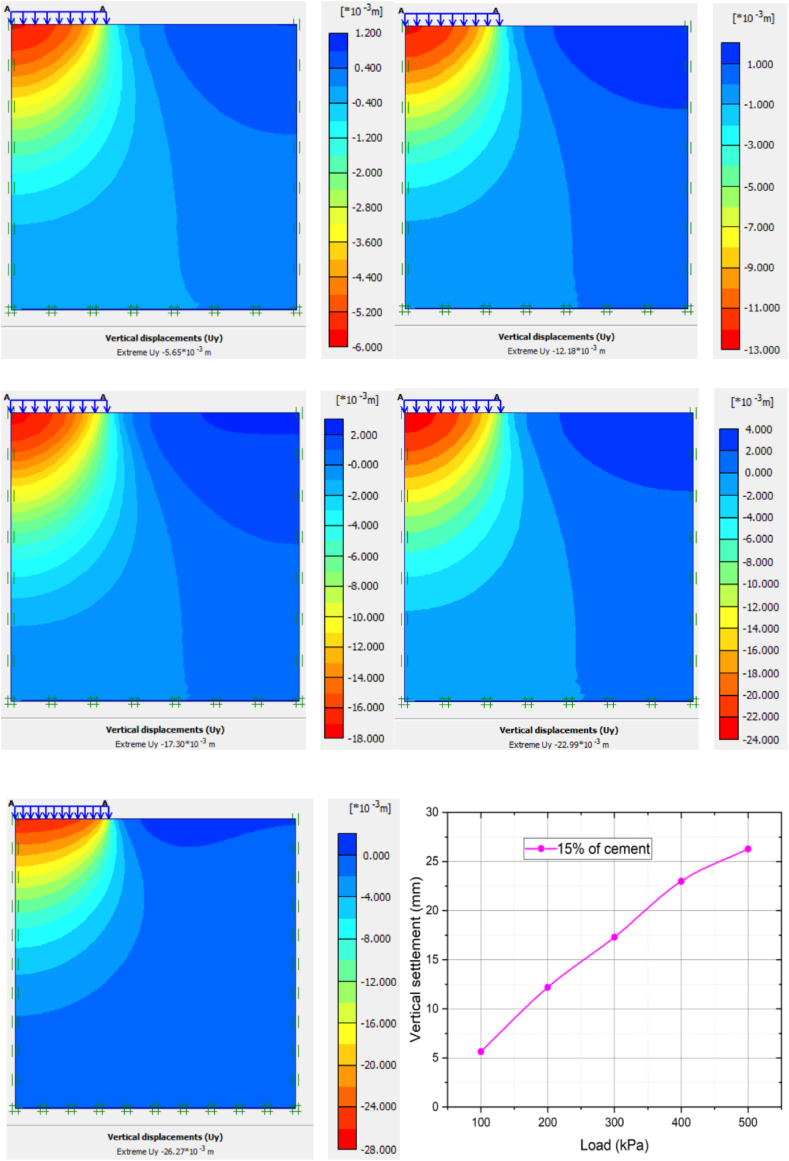
The Load-settlement curve of 15% of cement stabilized soft clay soil.

Figure 11 and Table 5 show relationship between load-vertical settlements of 15% of cement stabilized soft clay. The average settlement 15% cement modified clay soil for 100 kpa, 200 kpa, 300 kpa, 400 kpa and 500 kpa increased by 83.13%, 78.20%, 75.74%, 75.01%, and 78.91%, respectively. The settlement value was less compared to 9% and 12% of cement stabilized clay soil. This indicates the improvement of settlement of clay soil is significant at the optimum percentage of 15% cement. It is also clearly shown that the graph is increasing in linear scale with low values due to soil-cement reaction and high bearing capacity value.

### 4.1 Validation of finite element analysis

**Figure 12:**
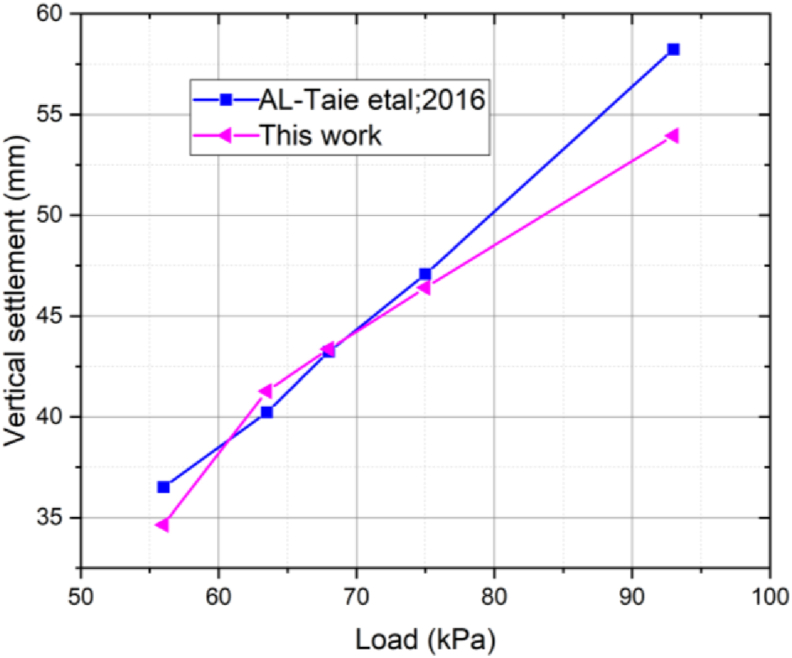
Validation of the FEM results.

Figure 12 presents the validation of the soil model with literature. The finite element analysis for the soil geometry of raft foundation (25 × 60) m under various added loads of 56, 63.5, 68, 75, and 93 kPa studied using the hardening soil model. The computational geometry, the applied loads and the soil model are similar with [30]. However, the input parameters used in hardening soil model for the simulation are obtained from the experimental result of this work. As a result, the vertical settlement of this work is in good agreement with the literature.

## 5 Conclusions

The study was successfully performed and showed that cement stabilization of soft ground increases shear strength and reduces deformation. Mechanical particles size analysis showed that more than 85% of the total mass passes through a sieve size 75 μm. this indicates that the soil is classified as fine-grained soil. From hydrometer analysis, more than 45% of the particle size of most samples is less than 5 μm this indicates that this soil type in the study area are strongly influenced by presence of clay fractions. The pre-consolidation pressure increased with increasing stabilizer content. The result of pre-consolidation pressure test ranges from 190 kPa to 340 kPa and the optimum value is at 15% of cement. The compression and swelling indices of untreated and treated soils are varied from 0.33 to 0.07 and 0.069 to 0.016, respectively. This indicates decreases in the compressibility index and swelling indices as cement percentage increases.

Based on the results of Plaxis 2d, we investigated the vertical displacement of natural soft clay by adding various loads in a finite element analysis. The result shows that the last settling value did not increase linearly. This mainly indicates that the soil was unable to receive this load and was very compacted. For 9% cement-stabilized soil, settlement increases with increasing load, but the magnitude of vertical settlement at the final applied load is 81.68 mm compared to natural soft clay soil. 12% of cement-stabilized soft clay soil had lower settlement values than 9% of cement-stabilized soil. This indicates that as the proportion of cement increases, the settlement value decreases. The settlement seen in untreated soft clay was 124 mm. The sedimentation of 15% cement is 26.77 mm. Improvement of soft clay soil with 15% cement is observed to further improve subsidence. There was a big difference in the size of the untreated and treated soft ground. This is the result of the soil cement reaction, and the curing time of the sample also affects the results. Pressure bulb of finite element analysis shows that the stress counter or a line which connects all the points below the ground surface at which the vertical pressure is the same. Pressure at points inside the bulb greater than that at point on surface of the bulb and pressure at point outside the bulb are smaller than that value. The pressure bulb indicates that, a flexible footing rests on cohesive soil, the settlement of the soil at the edge is minimum, but maximum at the center of footings. As the depth of soil layer increases, the settlement intensity decreases. The square footings load placed on the soil mass will induce stress within the soil. It is concluded that from pressure bulb diagram, while depth increases the deformation reduced to negligible value.

## Declarations

### Author contribution statement

Damtew Tsige and Meaza Korita: Conceived and designed the experiments; Performed the experiments; Analyzed and interpreted the data; Contributed reagents, materials, analysis tools or data; Wrote the paper.

Adamu Beyene: Conceived and designed the experiments; Analyzed and interpreted the data; Contributed reagents, materials, analysis tools or data.

### Funding statement

This research did not receive any specific grant from funding agencies in the public, commercial, or not-for-profit sectors.

### Data availability statement

Data included in article/supp. material/referenced in article.

### Declaration of interest's statement

The authors declare no conflict of interest.

### Additional information

No additional information is available for this paper.
